# Rage against the authority machines: how to design artificial moral advisors for moral enhancement

**DOI:** 10.1007/s00146-024-02135-3

**Published:** 2024-11-30

**Authors:** Ethan Landes, Cristina Voinea, Radu Uszkai

**Affiliations:** 1 Department of Philosophy, University of Zurich, Zürichbergstrasse 43, 8044 Zurich, Switzerland; 2 Uehiro Oxford Institute, University of Oxford, LittleGate House, 16-17 Saint Ebbe’s St, Oxford, OX1 1PT UK; 3 Research Centre in Applied Ethics, University of Bucharest, Splaiul Independenţei No. 20, Bucharest, Romania

**Keywords:** Artificial intelligence, Moral enhancement, Artificial moral advisors, Moral advice, Deference

## Abstract

This paper aims to clear up the epistemology of learning morality from artificial moral advisors (AMAs). We start with a brief consideration of what counts as moral enhancement and consider the risk of deskilling raised by machines that offer moral advice. We then shift focus to the epistemology of moral advice and show when and under what conditions moral advice can lead to enhancement. We argue that people’s motivational dispositions are enhanced by inspiring people to act morally, instead of merely telling them how to act. Drawing upon these insights, we claim that if AMAs are to genuinely enhance people morally, they should be designed as inspiration and not authority machines. In the final section, we evaluate existing AMA models to shed light on which holds the most promise for helping to make users better moral agents.

Recent breakthroughs in the field of artificial intelligence (AI), such as the development of large language models (LLMs), renewed hope in the project of moral enhancement. Already, there is growing interest in the development of artificial moral advisors (AMAs), AI systems created for moral enhancement (Constantinescu et al. 2022) with several AMA models conceptualized thus far (Savulescu and Maslen 2015; Giubilini and Savulescu 2018; Lara and Deckers 2020; Lara 2021; Giubilini et al. 2024). Briefly, AI-based moral advisors could help in the selection and processing of relevant information for decision-making, they could make users aware of the biases and inconsistencies that plague their reasoning and warn them of potential cognitive and moral limitations. Moreover, AMAs can be trained on vast databases of philosophical works on moral and ethical theory and can offer advice based on the texts processed. In other words, AMAs would act as personal assistants, with access to vast amounts of information relevant to moral decision-making, able to correct people when they are on the verge of making mistakes that go against their declarative values and offering advice in moments of impasse.

Still, morality is not an algorithmic business. Being a moral agent presupposes engaging in specific practices “under the right conditions and with sufficient opportunity for repetition” (Vallor 2015, 109).^1^ There is no single algorithm that can be applied across all contexts to guarantee moral action, as the situations we find ourselves in vary, requiring different types of moral reasoning attuned to the morally relevant features of each situation. Therefore, to act morally, agents must invest “considerable interpretive effort and discerning attention to the morally salient features of the given situation” (Vallor 2015, 113).

Although ‘interpretative effort and discerning attention’ belong to the sphere of intellectual (dianoetic), rather than moral virtues, there is a tight connection between the two. One cannot develop moral virtues without also developing intellectual virtues: “dianoetic virtues pertain to the rational part of the soul and are involved in the way ethical virtues are properly developed.” (Constantinescu et al. 2021, p. 808). More precisely, ‘interpretative effort and discerning attention’, while intellectual virtues, are crucial for moral action because they make moral deliberation possible. They enable agents to grasp the relevant context and make sense of the situation’s variables, facilitating moral behavior.

Instead, when relying on technology to solve moral problems that would otherwise require critical reflection and engagement, the worry is that the motivation, as well as the cognitive and emotional resources required to become better moral agents, will gradually weaken because they are not properly exercised. This would amount to moral deskilling (Vallor 2015, p. 111). In other words, by failing to exercise our moral skills because of relying on AMAs, we would, in the long run, become less competent moral agents than before the use of these technologies (although there is a possibility that we could still act morally). The question at stake here is not whether we end up doing the right thing by following the advice offered by the AMAs, but whether human beings become better moral agents using them. When someone intends to do something wrong but, by chance, ends up doing the right thing, it does not make them a morally good or virtuous person. Consequences matter less than moral motivation, at least from the moral enhancement angle, as we argue below.

While the potential of AMAs in fostering moral enhancement is promising, a detailed exploration of questions regarding the underlying epistemological mechanisms of moral advice and its impact on users’ moral motivation is still lacking. Our focus in this paper is to clear up the social epistemology of learning morality from AMAs to clarify when there is a risk of deskilling and when moral advice can lead to moral enhancement. We will examine not only how people can learn from AMAs but also how people should learn from AMAs. This will allow us to offer concrete guidance, driven by work in the social epistemology of morality, on how to build AMAs to lead to moral enhancement.

Section 1 starts with a brief consideration of what counts as moral enhancement and considers the risk of deskilling raised by machines that offer moral advice. Section 2 focuses on the epistemology of moral advice, and we introduce the distinction between deferring to something as an authority and using its input as a source of moral inspiration. We show that people’s motivational dispositions are enhanced by inspiring people to act morally, instead of merely telling them how to act. Drawing upon these insights, Sect. 3 argues that the way AMAs are designed is not neutral, insofar as AMA design can influence how users relate to it. We show that if AMAs are to genuinely enhance people morally, they should be designed as inspiration and not authority machines. In the final section, we evaluate existing AMA models to shed light on which holds the most promise for helping to make users better moral agents. In line with Lara and Deckers (2020) and Lara (2021), we argue that Socratic AMAs are the most plausible candidates for an inspiration machine that could lead to moral enhancement. By adopting a conversational stance that questions users, instead of telling them what to do, Socratic AMAs offer the possibility for users to cultivate or develop their moral motivational dispositions, thus bypassing the risk of deskilling.

## AMAs, moral enhancement, and moral deskilling

Human beings fall short as moral agents because of their inherent limitations (Giubilini and Savulescu 2018, p. 170). First, our information processing capacities are constrained by cognitive bounds, which hinder our ability to analyze all pertinent information relevant to making moral decisions. This could lead to suboptimal moral choices—e.g., I donate money to a charity without knowing how it uses the money. We also fail to appropriately integrate relevant information in decision-making because of emotional and gut reactions and biases, and this makes us suboptimal moral judges—e.g., I donate to a charity in my country as its causes move me, although my money would be better allocated elsewhere. Finally, even if we hold the relevant information and become aware of our biases, we might still fail to act morally because of the weakness of the will—e.g., I know that I should donate my surplus money to help others and I know that I should research what charity does the most good, but I do not do it as I keep spending my money on frivolous things.

AMAs can address these limitations in three ways: (a) they can unobtrusively gather and process information relevant to moral decision-making—either information about the environment or normative information—that people ignore due to their cognitive limitations; (b) they can analyze behavioral data to make users aware of their biases, patterns of reflection and action; (c) they can potentially offer users new perspectives on moral issues, some even unthought of by humans.^2^ AMAs could thus work as moral advisors that put people on the right moral track, by helping them improve their moral reasoning and overcome the limitations that stand in the way of moral behavior. Would this be enough for moral enhancement? To answer this question, we would first have to know what moral enhancement is.

### Moral enhancement

Raus et al. (2014) show how definitions of moral enhancement vary along several lines. First, there is contention regarding the methods of enhancement—some advocate for a broad approach where any means can achieve moral enhancement, while others emphasize the efficacy of specific interventions (biomedical, genetic, neural, etc.). Second, there is debate over the enhancement target, and whether such interventions should focus on individuals or humanity as a whole. In addition, opinions diverge on the passive reception of enhancement versus active participation in the process, alongside discussions on the requisite effectiveness of interventions for them to qualify as enhancements.

Despite the diversity of definitions, a minimal conception of enhancement, which we also adopt in this paper, would require, among other things, which it would improve people’s moral motivations to act. For example, Douglas (2008, p. 228) argues that moral enhancement is any intervention that “will expectably leave the enhanced person with morally better motives than she had previously”. Similarly, Persson and Savulescu (2019, p. 7) claim that for something to be a moral enhancer, “it must enhance your moral motivation, your disposition to (decide and) try to do what you think you ought morally to do”. On this view, punishment or rewards for (im)moral behavior and mere conformity to moral norms or advice cannot, by themselves, make people better moral agents. They might end up doing the right thing because of fear of punishment or social opprobrium or because they want the rewards, but these mechanisms (punishment/rewards) do not always change people’s internal moral motivations (although there are cases when they might). On this minimal view of moral enhancement, the cause for moral action must be internal to the agent. Therefore, the view we adopt in this paper is that moral enhancement is accomplished by any means that modify motivational dispositions and lead to doing what we ought to do for its own sake (Persson and Savulescu 2019, p. 11).^3^

We call this a minimal view of moral enhancement because it brings out something common to most ethical theories: moral action involves a degree of self-sacrifice, understood as a constraint of one’s self-interest, to benefit others. Thus, “Increasing the willingness to sacrifice one’s interests for the benefit of others is a moral enhancement, on any account of morality” (Persson and Savulescu 2019, p. 108). Therefore, minimally, moral enhancement is any intervention that improves people’s motivational dispositions to focus not only on their interests and well-being but also to take into consideration others’ interests, even if this sometimes presupposes self-sacrificing.

One could ask what is the purpose of enhancing moral motivation, and this is where different ethical theories would answer differently. For example, virtue ethicists would say that enhancing moral motivation supports the practice of virtue and achieving eudaimonia, while deontologists would note that it helps agents to make the right moral choices. These questions are beyond the scope of this paper. The view that we take here on moral enhancement is a minimal one showing that for something to count as enhancement it must improve people’s moral motivations. On this view, would moral advice be effective in morally enhancing people?

### Deskilling and the problem of deference

Critics stress that overreliance on devices, especially on ‘smart’ tools, can lead to a loss of cognitive and moral skills (Howell 2014; Vallor 2015; for a review, see, also Danaher 2018, p. 630). This skepticism usually comes under the form of the “deskilling argument”, which posits that overreliance on external support for a particular task can erode individuals’ abilities to accomplish that task independently. This is because when relying on external support, in our case, AI assistance, people would not exercise their skills anymore given that tasks are resolved for them and, in time, those skills would gradually disappear. Take the example of constantly using GPS maps and AI assistance for orientation: this leads to the loss of orientation abilities (Dahmani and Bohbot 2020). It could be the case that deskilling in some domains (e.g., orientation), leads to upskilling in other domains (e.g., using digital devices), as instead of putting time and effort into trying to orient myself, I could simply develop a new, more useful skill, such as the efficient use of digital devices that would help me accomplish the task of orientation. But skills can be instrumentally or intrinsically valuable. Unlike the skill of reading a map, which is only instrumentally valuable, moral skills are intrinsically valuable, as they are conducive to virtuous characters (Vallor 2015, p. 112). But even in situations where moral skills do not crystallize in virtues, they are still intrinsically valuable as they are an integral part of what it is to be human. Therefore, if moral deskilling leads to proficiency in other areas, say, cooking or driving, the gain of other skills cannot compensate for loss of our ability to reason and judge morally. As Vallor warns, if we lose our moral skills, “we would be diminished as creatures were we utterly helpless to act justly and compassionately without their assistance” (Vallor 2015, p. 113).

Returning to the AMA case, if these systems dispense advice without showing *why* a specific recommendation was selected, there is a risk that users might unquestioningly adopt the advice provided by AMAs. Even if users would act morally by following the advice, they would not be morally enhanced, as their motivational dispositions would remain the same. What is more, in the case of decoupling (that is, in case of not being able to consult the AMA anymore) users will return to the previous moral states and will not be able to continue to do the right thing, because they never gained moral understanding using these AMAs. In other words, AMAs that just offer advice, without explaining why, could lead to moral deskilling, as there is a possibility people could rely on them and act on their advice, without thinking about the advice they are offered or understanding *why* that specific recommendation was made. AMAs could thus decrease the opportunities people have to exercise their moral skills, which in the end could potentially lead to moral deskilling.

Deskilling in the case of overreliance on the moral advice offered by AMAs stems from the risk of people forming beliefs based on deference on moral matters (Howell 2014, p. 389). Deference refers to what contributes to the formation, but most importantly, to *sustaining* beliefs. In the following section, we delve deeper into the concept of deference, elucidating its nature, the contexts in which it emerges, and showing why moral deference might prove detrimental to the deferring individual. In addition, we draw comparisons between deference and inspiration, exploring their respective impacts on moral enhancement. This groundwork will underpin our subsequent discussions on the optimal design of AMAs to promote moral enhancement.

## Authority vs. inspiration

There are two fundamentally different ways we learn from other people—by *inspiration* and by *deference* (Grice 1957; Hills 2020; Landes 2023)*.*^4^ To illustrate the difference, suppose Sonja and Rahij are trying to decide whether to go for a walk. While discussing it, Rahij notices that it has started raining. Rahij could convey this new information to Sonja by telling her “it is raining”, at which point, because Sonja trusts Rahij and thinks Rahij has his wits about him, takes Rahij at his word. Sonja *deferred* to Rahij that it is raining. Alternatively, Rahij could instead point out the window, getting Sonja to turn around and see for herself that it is raining (Grice 1957). Rahij showed Sonja that it was raining. In both cases, the end result is the same—Sonja knows it is raining and has gained a reason to not go for a walk. However, in one case Rahij is acting as an authority (albeit one whose authority is not particularly impressive), in the other Rahij is acting as a source of inspiration by pushing Sonja to learn something for herself that he already knows.

The distinction between learning by deference and learning by inspiration is found everywhere. A science teacher can tell their students that earthworms prefer cold, damp places to warm, dry places, or the teacher can push students to learn this behavior for themselves by setting up an experiment. An investor could tell their financial partner that one of their investments is doomed to bankruptcy or give their partner a knowing look while handing them an incriminating balance sheet. A moral philosopher could tell a student that one should not use others as a mere means to an end, thereby acting as a moral authority, or they could inspire the student to come to the conclusion themselves by having the student critically evaluate arguments and thought experiments (see Landes 2023).

When we learn by deferring to authority, or as it is usually called by epistemologists, learning via testimony (e.g., Lackey 2008; Sliwa 2012; Ranalli 2020), our knowledge relies on the speaker. Insofar as we know what an authority tells me, it depends on their actual or perceived expertise, authority, or reliability. If we find reasons to doubt the putative authority’s actual authority—e.g., they reveal themselves to be untrustworthy or it turns out their degree was fake—then we gain reasons to doubt anything we “learned” from them.

In contrast, when someone inspires us to learn something, a speaker causes us to learn it, but we do not depend on them in the same way as deference. Instead, our insight depends on our own abilities because we learn by our lights. Whereas in cases of deference, the speaker acts as an authority, in cases of inspiration, the speaker acts as an enabler. The speaker has done most of the hard work finding the information or idea of interest, by, for example, learning about the behavior of worms, by acquiring and reading the balance sheet, by developing the philosophical arguments themselves. Nonetheless, they leave it to the listener to connect the final dots themselves. Whereas the value of deference comes from providing a listener with knowledge based on someone’s unique expertise or experience, the value of inspiration comes from drawing a listener’s attention to a promising idea or bit of evidence and encouraging them to think by themselves.

Deference and inspiration have different advantages. By deferring to others, we can learn things well beyond our own abilities to check for ourselves. We can know that Constantine XI was the last emperor of Byzantium despite not being alive in the fifteenth century. Similarly, deferring to authority can teach us things that we lack the patience, desire, or expertise to learn for ourselves. We can know why the sky is blue—that the atmosphere scatters light a certain way—without spending the years necessary to gain enough knowledge about physics to check for ourselves. In contrast, what we can learn from inspiration is limited by our patience, knowledge, and abilities. We cannot check with Constantine XI whether he was in fact emperor when Constantinople fell, and to most of us, achieving an advanced degree in chemistry or physics sounds tedious.

For the purposes of discussing AMAs and moral enhancement, deference has two key disadvantages in comparison to inspiration. First, someone can learn via inspiration even when a speaker has no idea what they are talking about or are speaking insincerely.^5^ This includes instances like bullshit, which some have suggested AI output amounts to (Rudolph et al. 2023, Herzfeld 2023). Suppose Sonja is a biologist writing up the results section of her new paper. Sonja and Rahij have a running joke where any time they run into each other in the hall of their department building one says to another “I read your new paper, you really messed up the stats”. While getting coffee, they see each other and Rahij makes the joke. Upon hearing the joke, Sonja thinks back to her draft and realizes that she did in fact make a mistake in her analysis. Sonja learned something about her research upon hearing Rahij’s joke, even knowing it was a joke. Rahij unintentionally inspired Sonja.^6^ Similarly, LLMs or other forms of AI do not need to, properly speaking, believe their output or utter their output sincerely in order for us to learn things from them. We can be shown moral truths by AI (or any semi-animate or inanimate object) via inspiration without having to worry about the intentions, representational states, or expertise of the thing we are learning from.

The second disadvantage of deference from the point of view of AMAs and moral enhancement is that it is inconsistent with the exercise of practical wisdom, that is, with being a genuine moral agent (Crisp 2014). The reasons for this are varied (see Fileva 2023 for a recent critical overview), but for present purposes, we will focus on three varied reasons, an epistemic reason, a social or communitarian reason, and a virtue-based reason.

The first, epistemic, reason one should not defer to authorities for matters of morality comes from the fact that deference is limited in its ability to spread understanding. Generally, epistemologists distinguish between knowledge and understanding (Grimm 2012). Knowledge is taken to be belief-based and about factual matters. Someone may know that Constantin XI was the last emperor of the Byzantine Empire because they justifiably and correctly believe the fact. In contrast, understanding involves the grasping of how facts are related to one another. Someone could memorize any number of facts related to the collapse of the Byzantine Empire, but if they do not “see” how the facts are related, they do not understand why Constantinople fell. Understanding, including moral understanding, has the key benefit of being generative. If we understand *why* something is wrong—as opposed to merely knowing that something is wrong—we are better able to approach novel scenarios (Hills 2020). Because we understand why certain things are right or wrong in one situation, we can expand our knowledge to new cases.

As Hills (2020) argues, the value of understanding is related to learning by authority vs inspiration because while we can gain knowledge by deferring to authorities, we ultimately have to form an understanding on our own. While a historian might tell us about the rise of the Ottoman Turks in the thirteenth century, the sack of Constantinople during the Fourth Crusade, and many other key events during the decline of the Byzantine Empire, to understand why Constantinople fell, we need to connect the dots in our head ourselves (Grimm 2012). Similarly, Hills argues, while we might be able to learn through authority that we should not steal from a friend, if we do not understand why stealing is wrong, we will lack the ability to adequately approach future cases involving theft. Thus, if a moral teacher really wants us to grow, they should help inspire us to understand why something is right or wrong as opposed to merely relying on their authority to tell us that something is right or wrong. This is because even if the teacher is a reliable and trustworthy moral expert, by deferring to their advice we would not grasp the moral reasons backing the advice, we would not be able to justify ourselves to others and implicitly, we would not be able to reliable act rightly because we did not develop our moral sensitivity (Hills 2020).

A second reason we should not rely on moral authorities has to do with our duty to others in our community. Fileva (2023) argues that we should not defer to people on moral matters in many—but not all—cases because “everyone is assumed to have a duty—and not simply a right—to exercise one’s moral reasoning capacities” (p. 730). Fileva rejects the individual-focused reasons given by other authors, instead arguing the reason we should not defer to people on moral matters has to do with the duty we owe our larger moral community. We are members in our communities’ collective effort to be more moral as well as our society’s collective moral governance, and if we do not employ our own moral compass, we are free-riding on the work of others. Not only are we, Fileva argues, more likely to come to moral truth if we act collectively instead of deferring to some moral authority, but if we do not use our own moral compass to question our moral authorities—such as philosophers, priests, or our parents—we risk leading less moral lives by following the advice of someone who themselves is wrong or misguided.

Pessimism about moral testimony has also come from the direction of virtue ethics. Broadly speaking, virtue ethics is a type of account of morality that emphasizes acting well or acting with virtue over doing the right action (see Stohr 2006). The important feature of virtue ethics for the discussion of inspiration vs testimony is that acting virtuously requires doing actions for the right reason and developing the right sort of virtuous character (Crisp 2014). Howell (2014) argues that taking moral advice by testimony allows someone to act virtuously but prevents the person from developing their own virtue (see also Hills 2020). Virtues are thought to require development and practice, much like learning an instrument or a foreign language. By deferring to others’ moral advice, we are eliminating the opportunity to develop our own virtue and moral character for the sake of expediency. Crisp (2014, p. 130) calls this the phronetic argument: deference or relying on others for moral advice is in tension with “the value or aspiration of moral thought”; in other words, deference is incompatible with practical wisdom. More specifically, from a virtue ethics perspective, taking moral advice via testimony is the equivalent of skipping leg day at the gym. Howell also highlights how acting on the basis of testimony is problematically insincere. Becoming a vegan solely because our philosopher friend tells us that that is the right thing to do means that we lack the appropriate moral character to appreciate the wrongness of our actions. Thus, while outwardly, our behavior may appear virtuous, it is not actually arising out of our virtues, because we do not have the right dispositions that lead to the right moral action.

In what follows, we argue that AMAs could enhance moral motivation, thus serve as moral enhancement, if they inspire change, rather than resort to mere directives or coercion which encourage deference, or, in Lara’s words if they are “aimed at increasing the individual’s capacity to reflectively decide for themselves, rather than at directly influencing behaviour” (Lara 2021, p. 41). Therefore, the focus of an AMA should be on motivating people to become better moral judges and decision-makers. To accomplish this, AMAs should be designed as inspiration machines that motivate individuals to reflect on their behavior, guiding them toward developing character traits that enhance their moral reasoning and decision-making abilities, thus improving their moral dispositions.

## What do we want out of AMAs?

As previously discussed, if our aim with AMAs is to foster moral enhancement rather than moral deskilling, then AMAs must strive to enhance people’s moral motivations. The insights highlighted in the preceding section suggest that AMAs ought not to be fashioned as moral authorities that merely dispense advice under the form of directives, but rather as sources of moral inspiration. The way AMAs are designed is not neutral and can influence, to a great extent, how users relate to them, as we show in what follows.

To this end, let us contrast two ways of designing the social epistemic interaction between AMAs and their users, authority machines and inspiration machines. Authority machines are designed to produce moral advice that is meant to be taken literally, at face value, and as concrete guidance by users. Here, the advice primarily takes the forms of assertions about either moral principles—e.g., “always prioritize equality”—or specific actions—e.g., “you should order the vegan pizza instead of the steak”. The goal of any exchange between the AMA and the user would be to give the AMA enough information to generate advice appropriate for the situation, and a primary goal of the AMA’s design would be to encourage users to follow any advice to the letter.

Authority machines face several significant problems. Speaking practically first, authority machines require establishing expertise or trust in the users. Not everyone earns our respect as an authority, whether moral or otherwise. We will not order the vegan pizza merely because a self-styled prophet tells us that is what their god has declared is right. Some users may readily accept AMAs as moral authorities, as many users of LLMs appear to treat their outputs as authoritative, going as far as using LLMs to write legal briefs and filing the briefs without checking the accuracy of the outputs (Armstrong 2023). This is especially worrying in the case of AMAs that are preprogrammed with the users’ moral values and hierarchy of values (which is one of the AMA models advanced, as discussed in the next section) because people generally trust their moral values to be the superior to the ones others have (Tappin and McKay 2017) and are reluctant to change them. Therefore, if the machine is programmed with their values, and if the machine is seen as reliable (Lara 2021, p. 42), then it is not unreasonable to assume that people will treat these AMAs as authority machines. Moreover, by providing advice as a settled fact, authority machines encourage laziness and complacency on the part of the user. When surprising advice is generated or advice is given to resolve a moral puzzle the user faces, there is no reason for users to reflect on the AMA’s advice, if the AMA is used as an authority machine. This is because users would not see the rational connections between the reasons for the particular piece of advice offered and the advice itself (Lara 2021, p. 42). Thus, the design fails to encourage moral growth beyond merely forming standalone moral beliefs.

In contrast to authority machines, inspiration machines aim to produce moral advice that help users make up their own mind on moral matters. The goal is not to give moral directives but rather to help guide users through moral decision-making and help them understand and develop their own moral compass. Thus, inspiration machines are meant to help agents gain moral understanding and flourish as virtuous agents.

The most obvious form inspiration machine output could manifest is through open-ended questions—“why might you choose the vegan pizza over the steak?”. However, the form AMA output takes is just as important as the *stance* the AMA cultivates in the user towards the AMA output. Inspiration machines only function as inspiration machines if users see the output as something to be grappled with. Thus, just as a moral authority such as a hypothetical authority machine can assert a moral truth via a pointed question (see Hawley 2010, p. 398)—“are you *sure* you want to order steak instead of vegan pizza?”—an inspiration machine can inspire the user to think through a puzzle by asserting (or apparently asserting) something. An inspiration machine may help users learn by connecting dots between the user’s own thinking—“you are answering in line with the doctrine of double effect”—or bits of theory to ponder over—“the doctrine of double effect is the idea that …”. If the user genuinely engages with the inspiration machine as merely a source of inspiration, the output can even include situation-specific claims to be thought about and accepted or rejected. The (apparent) assertion “eating steak is the right thing to do because it will bring you the most pleasure” makes one common reason for ordering meat over vegan options explicit and may accordingly help people deconstruct and learn about their own moral decision-making.

The main practical challenge of an inspiration machine is cultivating the correct user stance towards the AMA. One aspect of this is motivational. Thinking through moral matters is difficult and cognitively draining, and being confronted with your own moral inconsistencies is uncomfortable or even infuriating. But it could also be rewarding since an examined life can be more fulfilling than one that is lived by other people’s lights. Blindly taking authoritative moral advice, in contrast, is easy because it offloads all of the cognitive, intellectual, and emotional efforts of moral deliberation. The other challenge towards designing an inspiration machine is similar to the challenge an authority machine faces in establishing trust or authority. What an inspiration machine needs is for its outputs to be considered by users as worth considering. That is, users need to see it as worth their time and effort to engage thoughtfully with the AMA.

To consider the importance of legitimacy for speech acts to become the basis of inspiration, return to Sonja and Rahij. Suppose Sonja is working on a new paper that includes data collection. Unbeknownst to her, Sonja has messed up her statistical analysis. Rahij sees her at the office coffee machine and jokes, as he always does when she is writing a paper, “Did you check you ran the right statistical tests?”. Sonja laughs and thinks nothing of it. Later she runs into Billy, a colleague with extensive training in statistics. The other week they had a discussion about when to use variations of various common statistical tests. Billy, knowing Sonja was working on a new paper says “Did you check you ran the right statistical tests?”. Why might Sonja justifiably react to Billy’s statement differently and take what Billy says as inspiration to go through the effort to check for herself whether she in fact made a mistake? In short, Billy’s question has legitimacy whereas Rahij’s question does not. Rahij is just joking, so his question does not have any obvious value in following up. Billy’s question comes from a place of expertise and sincerity, so Sonja has clear reasons to take the underlying advice—check your tests—seriously.

An inspiration machine, therefore, needs to be designed to thread the needle between two competing extremes. On one hand, it cannot be seen as an outright moral authority because then users will not grow as virtuous moral agents. They may instead deskill and become dependent on the AMAs moral decision-making, forgoing the effort required to grow. On the other hand, inspiration machines cannot be seen as merely guessing or making half-hearted attempts at growth. If the AMA lacks legitimacy, advice will not be sought by users or it will be met with the same dismissive eye roll we reserve for known bullshitters.

## Artificial moral advisors

In what follows, we evaluate models for designing AMAs already put forth in the literature against the authority/inspiration distinction drawn above. The purpose is to shed light on which AMA models hold the most promise in inspiring individuals to grow into better moral agents by enhancing their moral motivational dispositions. To that end, we endorse the Socratic model of AMAs developed by Lara and Deckers (2020), arguing it has the potential to lead to moral growth.

### Exhaustive and auxiliary AMA

The first, and possibly the most contentious model of enhancement through AI, is derived from machine ethics, where in the future AIs are imagined “to be capable of human-level moral decisions independently of human guidance.” (Liu et al. 2022, p. 436) and is referred to by Lara and Deckers (2020) as exhaustive AMAs. Within this paradigm, the focus is not on how we can build AIs that lead to moral enhancement, but rather how to build AIs that could become better moral decision-makers than humans would ever hope to be. For example, Dietrich (2001) argues that artificial agents will become vastly superior in terms of moral reasoning to human agents, because of their capacity to be impartial and to process all the relevant information for decision-making. For Dietrich, this means that we should allow these entities to multiply and finally overtake humans, given their vastly superior morality (Dietrich 2001, p. 531). Not every proposal for exhaustive AMAs is this blue-eyed about the potential of AI in moral decision-making, however. For example, Gips (1995) is not so pessimistic about human nature, and only suggests that the tirelessness, lack of emotions, impartiality, and consistency inherent in machines could potentially make them more adept moral reasoners than humans. Therefore, instead of human beings being made redundant by superior machines, Gips argues that human decisions should be guided by these machines that lack all of the human biases that plague moral decision-making.

By offloading all moral decision-making onto the AMA, exhaustive AMAs are the purest form of authority machines. Humans would have to follow the AMAs’ moral guidance without question or consideration. Previous authors have argued that exhaustive AMAs are degenerative (Lara and Deckers 2020; Volkman and Gabriel 2023), because the passive stance encouraged by exhaustive AMAs could potentially lead to a gradual erosion of human moral skills, ultimately resulting in a complete atrophy of capacities for moral judgment and behavior. Even if by deferring to the moral advice of these super-human moral AIs, users would end up doing the right thing, it would not be a case of moral enhancement, as people’s moral motivations would not be enhanced. What is more, when the machines and the humans would be decoupled, they would be no better off than before they started using these machines. While users of exhaustive AMAs might be more likely to do the right thing (under the very questionable assumption that the AMA is programmed to follow the correct moral code), the actions would not be sparked by people’s moral dispositions. In other words, exhaustive AMAs leave people’s moral dispositions intact and might even weaken them, so it is difficult to argue that they could lead in any meaningful way to moral enhancement.

A less extreme model is that of auxiliary AMAs (Savulescu and Maslen 2015; Giubilini and Savulescu 2018)*.* Savulescu and Maslen (2015) and Giubilini and Savulescu (2018) suggest very similar notions of AMAs that monitor users’ environment to alert “the agent to features of his own physiology, mental states or environment […] that might impair moral judgment and/or behaviour” (Savulescu and Maslen 2015, p. 84). The idea behind these two models^7^ of AMAs is that the user would start by inputting their own value hierarchy (Savulescu and Maslen 2015) or would choose a value framework (Giubilini and Savulescu 2018) and the AMA would use these bases to formulate its recommendations. In addition, the AMAs would observe users’ behavior and alert them of potential biases and other limitations that might distort moral reasoning, as well as warn users of the targets they aim for and that they might miss if they do not act in a particular way (Giubilini and Savulescu 2018, p. 174).

Auxiliary AMAs offer users a more active role in moral deliberation compared to exhaustive AMAs, but still not active enough to lead to enhancement. In both Savulescu and Maslen’s version (2015) and Giubilini and Savulescu’s model (2018), the AMAs are effective only for individuals already aligned with the right moral values. This is because the AMAs would only recommend courses of action that accord with the value hierarchy or framework previously inputted by users. These AMAs do not push people to reflect on or question their values, which points towards the fact that they do not provide the impetus for gaining moral insight that leads to an enhancement of moral motivation. What is more, because these AMAs use users’ moral outlooks as a basis for the recommendations they dispense, they encourage deference to their advice and, implicitly, encourage laziness and complacency on the part of the user, as people already tend to see themselves and their moral values as superior to those of others (Tappin and McKay 2017), thus they are nor very likely to rethink or even completely reconsider them. Users might also feel that their moral work is done when configuring the AMA, thus there is a probability that they might defer to the AMAs given that they believe they understand the reasons AMAs have to offer that particular piece of advice. What is more, in cases of divergence between the advice provided by the machine and the users’ moral values, the users would have to either defer to the machine or refuse its advice: “As the agent does not need to understand the rational connections between their values and the decisions that are made by the system, their moral skills may not be enhanced a great deal.” (Lara and Deckers 2020, p. 281). Once again the problem of decoupling arises: if the AMA stops working, the agent would not be better off morally than before using it.

While existing on a spectrum, both exhaustive and auxiliary AI enhancements share a common assumption: individuals are likely to make improved moral choices by outsourcing either all or some essential aspects of moral deliberation to an AMA (Volkman and Gabriels 2023, p. 8), thus relying on the AMA to serve as a moral authority that tells them what they should do. Auxiliary AMAs assume that users are already fully mature moral reasoners who, at most, struggle with the odd failure of self-control or to infer the consequences of their known moral principles. But as we learn about our moral compass and the world, we are sometimes confronted with perspectives or considerations that radically change our moral perspective. Giving authoritative moral advice based on principles we had selected in the past may help us do more good overall, but it does not enhance our moral motivation or help us develop our moral virtue and understanding.

By telling people what to do, irrespective of the extent to which user-inputted data contributes to the final outcome, the bulk of the moral decision-making burden falls on the AMA. If the AMA does not offer reasons for its recommendations, then it operates as an authority machine, offering little inspiration for the enhancement of moral motivation. Relying on such tools for moral decision-making may yield limited effects in terms of enhancing morality, particularly if we conceptualize moral enhancement, as we do in this paper, as the enhancement of individuals’ motivations and character traits leading to moral actions. Analogous to how drivers delegate most decisions to navigation apps like Google Maps or Waze do not learn their way around an unfamiliar environment as quickly or effectively as people who do not use such apps (see Howell 2014), exhaustive and auxiliary AMAs could improve users’ moral behavior but would not count as enhancement as they would not improve users’ moral dispositions.

### Socratic AMA

A third category of AMAs, advanced by Lara and Deckers (2020) in response to the shortcomings of previous proposals, is the Socratic one. A recent proposal from Lara and Rodríguez-López (2024, p. 9) builds on the Socratic AMAs by framing their outputs as Socratic nudges which could be judged on their ability to increase the “argumentative productivity” of the human user. The main idea is that the AMA would not work on a set of predefined values, either inputted by users or by various moral experts, but would aim only to help users reach better decisions. Thus, instead of deliberating in the place of users, the Socratic AMA would help them through decision-making processes. This shows that what is important here is the formative role of the AMA. In other words, “the aim is to help the agent learn to reason ethically” (Lara and Deckers 2020, p. 282). The Socratic AMA would accomplish this task by: (a) providing users empirical support for decision-making, thus helping with anchoring ethical judgments on an empirical basis; (b) improving conceptual clarity, as an AI-based AMA could potentially detect that users employ normative concepts without rigor, which can complicate and muddle moral reasoning; (c) helping users notice the logical inconsistencies of their arguments, which can, in the long run, better users’ capacities to apply argumentative logic; (d) detecting biases that lead to distorted moral judgments; (e) advising users on how to put into practice their decisions, by offering examples of people’s actions in similar situations. In a similar vein, Giubilini et al (2024) propose iSAGE, a fine-tuned LLM acting as a 'digital ethical twin' of users, that analyzes individuals’ ethical views and decision-making history to provide personalized, real-time moral advice. The purpose of iSAGE, which is supposed to complement other AMA models, such as the Socratic one, would be to “infer and explicitly express their occasionally changing values and preferences, thus helping with the acquisition of self-knowledge” (Giubilini et al. 2024, p. 54). iSAGE could thus help with sparking moral reflection on one’s past experiences, current values, and aspirational goals, encouraging introspection on how one’s choices align with their values and the kind of person they aspire to be. This process of guided self-exploration could empower individuals to make morally sound decisions and refine their understanding of themselves, which could be a path to moral enhancement. Notice, though, that just as in the case of the Socratic AMA, the role of iSAGE is to spark moral reflection, instead of merely offering ready-made moral advice. 

In contrast to exhaustive and auxiliary AMAs, the Socratic systems and iSAGE do not aim to prescribe actions by providing ready-made answers based on predetermined criteria. Instead, they seek to cultivate understanding of the rational force of arguments and of oneself through dialogue. Specifically, these AMAs would pose a series of questions, including inquiries like “Why? And why? What makes you think that? Is this your last reason? Why do you think this is the best reason? What about this other reason? What do you mean with this word? Do you know there are other meanings? Are you aware that your assertion has no scientific basis? Are you aware that both assertions are contradictory? Are you aware that this deduction/induction/analogy… is not valid?” (Lara and Deckers 2020, p. 284).

The Socratic AMA, through dialogue and questions, aims to spark users’ curiosity and enhance their awareness of the elements required for formulating sound moral arguments and engaging in effective moral reasoning and decision-making; iSAGE, on the other hand, is meant to help people know themselves better. So, the Socratic AMA, as well as iSAGE, work as inspiration machines that indirectly enhance people’s motivational dispositions by making them more sensitive and critical towards the positions they hold, as well as making them question these positions by pointing towards morally salient features of particular situations that would otherwise have been ignored. What is more, because of their dialogical nature that guides users through various moral justifications, these AMAs avoid the risk of being used as authority machines that instill deference. Unlike exhaustive and auxiliary AI enhancements, the Socratic AMA and iSAGE serve a distinct formative or pedagogical role with a primary focus on elevating the user’s skills. As a dialogical agent, made possible by LLMs, the Socratic AMA and iSAGE do not dictate users’ actions but instead demonstrates how to become more adept moral agents by honing their ability to reason morally, fostering sensitivity to moral contexts, and encouraging an open-minded approach to new perspectives. In essence, the Socratic AMA and iSAGE question individuals when their judgments lack consistency or are contradictory, serving as inspirational tools that enhance users’ practical wisdom. 

## Conclusions: what we should want from AMAs

LLMs are a distinctly new type of moral agent. They are potentially more knowledgeable than us about morally relevant facts while not being susceptible to the same biases and weaknesses of reasoning that we are. The question becomes, how should we design AI to lead to moral improvement? Already Lara and Deckers (2020) and Lara (2021), as well as Giubilini et al (2024) argued that the Socratic AMA, and iSAGE, are the most promising models of AI-based moral advisors for moral enhancement. In this paper, we supported their claims by bringing additional epistemic reasons that have not been comprehensively considered yet and by building a bridge between kinds of literature that have remained confined in theoretical silos: social moral epistemology, moral enhancement, and AI ethics.

Focusing on the epistemological underpinnings of the project of building AMAs to improve our moral lives and provide the preconditions for moral enhancement, this paper has sought to provide a new framework for assessing both existing and future versions of AMAs. First, both exhaustive and auxiliary AMAs fall within the boundaries of what we have called ‘authority machines’. Leaving aside the question of whether AI tools can be moral experts, such AMAs rely on epistemic deference which, in the moral realm, is problematic for at least three reasons: it falls short epistemically, it fails to hold up our place in our moral community, and it prevents us from developing our virtue. In short, deference does not lead to an enhancement of moral motivational dispositions.

If we agree that the purpose of moral enhancement interventions is to improve our skills to reflect and our ability to act morally by improving moral motivational dispositions, then any interaction with an AMA that does not provide moral understanding would be ineffective. This, however, is the biggest drawback that authority machines have. In contrast, inspiration machines like the Socratic AMA (Lara and Deckers 2020), or iSAGE (Giubilini et al. 2024), potentially paired with Socratic nudges (Lara and Rodríguez-López 2024), would contribute to moral enhancement simply by encouraging users to develop and exercise their moral skills. After all, unexamined moral advice is not worth following.

Designing authority machines seems, at least in principle, an easy task. Inspiration machines, on the other hand, would need to be built in such a way that their outputs are deemed by their users not as a source of knowledge, but as something worth considering*.* If they are deemed as a source of knowledge, then they are not inspiration machines and therefore would contribute to deskilling. For their outputs to be deserving of our attention, much work would need to be done to establish their legitimacy. One noteworthy difficulty of this project rests upon the fact that it seems to go against the prevailing social practice (and expectation) to build artificial assistants that simplify our lives and make choices easier.

## Data Availability

Not applicable.

## Footnotes

1 This holds for all moral theories, not only for virtue ethics. For example, applying Kant’s categorical imperative or utilitarian decision rules requires “considerable interpretive effort and our discerning attention to the morally salient features of the given situation” (Vallor 2015, p. 113). The same holds for utilitarianism.

2 For example, research of professional Go players found their playing improved after they observed how AI Go players “think” (Shin et al. 2021). The AI provided players with new strategies to play Go, and so AMAs may provide new ways of thinking morally.

3 It is interesting to note that even authors who are critical of the particular ways in which authors like Douglas, Persson and Savulescu argue that moral bioenhancement could be achieved do not deny the quintessential role that moral motivation plays for moral enhancement (for more details, see Jotterand and Levin 2019).

4 Please note the difference in terminology from Landes (2023) and Hills (2020) who use *transmission* and *propagation* and Grice (1957) who distinguishes between *telling* and *showing* or *getting someone to think*. This choice is stylistic and we do not believe anything hangs on it.

5 Lackey (2008) argues we can learn from testimony when we know someone is lying to us, but Lackey’s point is different from the point being made here. Lackey imagines a case where the speaker is so reliable at lying that we can know what is true by inferring the opposite of what they say. Our point here is instead that even in cases where a speaker’s utterance has no reliable connection to the truth at all, one can still learn via inspiration.

6 There does not even need to be a genuine speaker for inspiration, so for the purposes of this paper, it does not matter if you think AI’s outputs are genuine speech acts. Suppose instead that while Sonja is getting coffee, she thinks for a moment that she hears Rahij joke “I read your new paper, you really messed up the stats”, but what she actually heard was a colleague in their office messing around with a text-to-speech generator “saying” gibberish that happened to sound similar from afar. Even this can still inspire Sonja to spot a mistake in her stats.

7 The difference between the two models is that in Giubilini and Savulescu’s view (2018, p. 177) moral experts should pre-program the AMA, determining the “moral principles or constraints to be incorporated into the AMA as basic filters” (p. 178). In this way, the AMA could not be programmed to offer immoral or deviant advice.

## Code availability

Not applicable to this paper.

## Materials availability

Not applicable to this paper.
